# Functional Outcome of ACL Reconstruction Following Pre-reconstruction Rehabilitation vs. None Rehabilitation: A Systematic Review and Meta-analysis

**DOI:** 10.1055/s-0044-1779327

**Published:** 2024-04-10

**Authors:** I Gusti Ngurah Wien Aryana, Febyan Febyan, Dominicus Dimitri, Shianita Limena, Leonardus William Kuswara

**Affiliations:** 1 Departamento de Ortopedia e Traumatologia, Hospital Geral Prof Ngoerah, Faculdade de Medicina, Universidade Udayana, Bali, Indonésia

**Keywords:** anterior cruciate ligament injuries, anterior cruciate ligament reconstruction, pre-operative rehabilitation

## Abstract

**Objective** The aim of this study is to analyse the needs for pre-operative rehabilitation in patients undergoing ACL reconstruction.

**Methods** The database reports were searched within 2018 to 2023, using PubMed, Cochrane library database, Medline, and other published trials. A statistical analysis was made from Review Manager.

**Results** Pre-operative rehabilitation group shows significantly higher 2 years post-operative KOOS score in all subscore and the total mean of the score, pain (p < 0. 0001), symptoms (p < 0. 0001), ADL (p < 0. 0001), sports and recreations (p < 0. 0001), QoL (p < 0. 0001), and the total mean of the KOOS score (p < 0.0001). In contrary, pre-operative rehabilitation group shows insignificantly higher score on 3 months post-operative Lysholm score (p = 0.12).

**Conclusion** This meta-analysis conclude pre-operative rehabilitation may provide better long-term post-operative outcome, however it may not provide much of a short-term outcome. It is recommended to add pre-operative rehabilitation as a guideline for ACL injury management to improve long-term outcome of patients with ACL injury undergoing ACL reconstruction procedure.

## Introduction

ACL reconstruction remains the mainstay of treatment for ACL ruptures worldwide. The rate of ACL reconstruction keeps trending consistently with the growing number of ACL ruptures. 1 2 It could reach as high as 68,6 cases per 100,000 persons annually. 3 Lately, a more active and younger population has been affected. Thus the demands for quicker recovery and optimal knee functions are significant. 4 Researchers aim to obtain the best strategies for managing ACL rupture cases. 5

Though seems insubstantial; a rehabilitation program serves as an excellent supplementary treatment plan enhancing the better outcome of ACL reconstruction. 6 Eitzen et al. 7 mentioned a pre-operative quadriceps muscle strength produces better knee functional outcomes. In contrast to the none rehabilitative group pre-operatively, it yields less-satisfactory functional outcomes. 8 This study aims to answer the question “is pre-rehabilitation necessary to enhance the outcome of ACL reconstruction?” by comparing pre-reconstruction rehabilitation vs. non-rehabilitation with the functional outcome of ACL reconstruction through recent publication.

## Methods

This systematic review and meta-analysis were performed following PRISMA guidelines, QUOROM checklist, and flow diagram for meta-analysis for Randomized Controlled Trials (RCTs).

### Search Strategy and Selection Criteria

We performed a systematic search of English language literature on PubMed/MEDLINE, ZETOC, EMBASE, AMED, CINAHL, Cochrane Central Register of Controlled Trials (CENTRAL), and ClinicalTrials.gov, published from January 2018 to January 2023. Search terms include, but are not limited to, "pre-reconstruction rehabilitation,” “pre-operative rehabilitation,” “pre-operative exercise”, “pre-rehabilitation”, “none rehabilitation”, “anterior cruciate ligament”, “ACL rupture”, “ACL tear”, “treatment”, “quality of life”, “functional score”, “KOOS”.

All types of randomized controlled trials, cohort and case series published as a full article were included in this study. The pieces were selected based on the stated inclusion and exclusion criteria according to the PICO (Population, Intervention, Comparison, Outcome) method as depicted in Table 1 .

**Table 1:** PICO Table for Inclusion dan Exclusion Criteria

	Inclusion criteria	Exclusion criteria
**Population**	• Age 18-40 • Planned for reconstructive surgery • Attending pre-operative rehabilitation	• Age < 18 or >40 • Acute ACL tear • Complex injury
**Intervention**	Pre-reconstruction rehabilitation program.	• Patients undergo conservative treatment
**Control**	Only post-operative rehabilitation No rehabilitation program	
**Outcome**	• KOOS score • Lysholm score	• Outcome measures not reported in completion. • Outcome measures only report the difference. • Outcome measures collected at different. • Outcome measures are not comparable to one another
**Design**	Randomized Controlled Trial, Cohort, Case Series	Case Report, Systematic Review, Meta-analysis

### Data Extraction

The data were extracted using a standardized data collection form by a research team with each chosen article screened independently by two reviewers. Disagreements between reviewers regarding whether to include or exclude a study will be resolved by consensus and consultation with a third reviewer if necessary. Variables collected include the KOOS score and Lysholm score.

### Data Analysis

The extracted data were assessed for clinical heterogeneity. Due to the differences in exercise interventions investigated, study populations, and outcome measures, it was deemed that included studies were not homogenous. Thus, a meta-analysis could not be completed. The data analysis and forest plot generated were made by using Review Manager (RevMan), The Cochrane Collaboration, 2020

### Quality Assessment

Included studies were assessed in terms of quality by two independent reviewers based on the 13-item of the 2015 Updated Method Guideline for Systematic Reviews in the Cochrane Back and Neck Group.

## Results

**Fig. 1:**
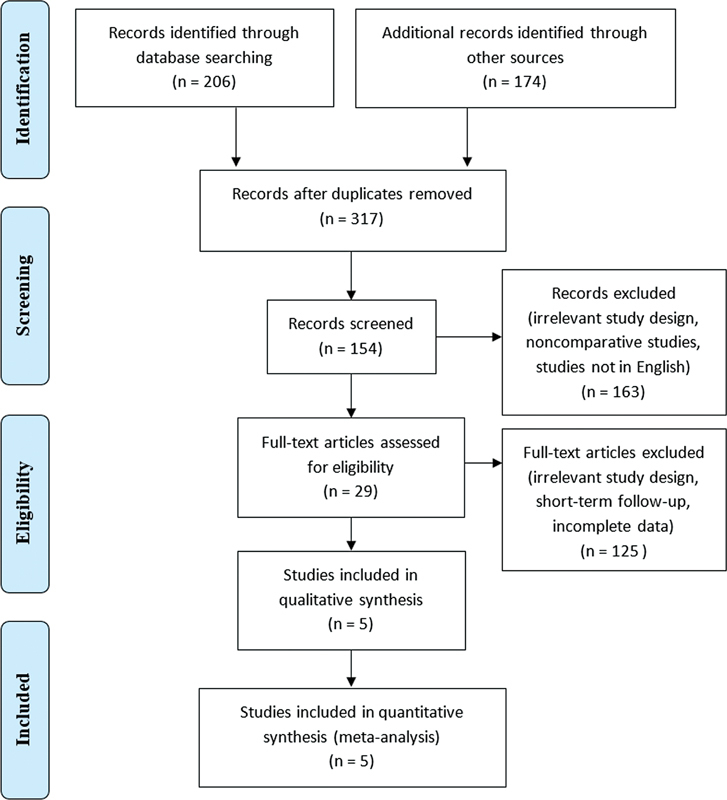
Research Screening Guided by the PRISMA Flow Chart.

The study selection process is presented in the PRISMA flow. Records identified through database searches are 206, while additional records identified through other sources are 174. Duplicates of 63 studies were removed. Records were screened by the research team and 125 records were excluded. Full-text articles assessed for eligibility of 29 articles. Full-text articles excluded due to design or data collection time point. Hence there are 5 studies included in qualitative synthesis ( Fig. 1 ). The included studies are presented in Table 2 . 5 9 10 11 12 Population characteristics of the included studies are presented in Table 3 5 9 10 11 12 while outcome characteristics of the included studies are presented in Table 4 . 5 9 10 11 12

**Table 2:** Studies included in the meta-analysis

No	Author (year)	Journal	Studi Design	Level of Evidence
**1**	Amaravati et al. (2013) 10	ISAKOS	Single blind prospective RCT	2a
2	Failla et al. (2016) 5	American Journal of Sports Medicine	Cohort	3
3	Grindem et al. (2015) 9	British Journal of Sports Medicine	Cohort	2a
4	Reddy et al. (2020) 11	Ambulatory Surgery	prospective randomised study	2a
5	Aiyanna, et al. (2022) 12	International Journal of Recent Scientific Research	Prospective study	2a

**Table 3:** Population Characteristics of the Studies

No	Author (year)	Intervention	Control	Sample size (n)	Gender (Male/Female)	Age (years)	Follow-up (months)
1	Amaravati et al. (2013) 10	R	NR	I: 32	NA	NA	ROM: 6w, 3m, 6m, 1y
				C: 31			Pain, Grith, Lysholm, Tegner activity, LEFS, IKDC, Hop test
2	Failla et al. (2016) 5	R	NR	I: 192	Male 55%	24,3 ± 10	IKDC and KOOS pre and 2 years post
				C: 1995			
3	Grindem et al. (2015) 9	R	NR	I: 84	Male 54%	24,7 ± 9	KOOS pre op (post rehab), 2 years post op
				C: 2690			
4	Reddy et al. (2020) 11	R	NR	I: 20	M: 38	I 27,48	IKDC Grade, Lysholm, ROM pre, 3w, 6w, 3M,6M post op
				C: 21	F: 3	C 28,17	
5	Aiyanna, et al. (2022) 12	R	NR	I: 15	M: 25	0-20: (27%)20-40: (67%)40-60 (6%)	IKDC, KOOS, Lysholm score3, 6, and 12 months
				C: 15	F: 5		

**Abbreviations: C** , Control; **I** , Intervention; **IKDC** , International Knee Documentation Committee; **KOOS** , Knee Injury and Osteoarthritis Outcome Score; **LEFS** , Lower extremity functional scale; **NR** , No Rehabilitation; **R** , Rehabilitation; **ROM** , Range of motion.

**Table 4:** Outcome Characteristics of the Studies

No	Author (year)	Conclusion	Group (N)	Period of Rehab	KOOS Mean	KOOS Pain	KOOS Symptoms	KOOS ADL	KOOS Sports	KOOS QOL	Lysholm Score
1	Amaravati et al. (2013) 10	Pre op Exercise resulted in better Post op functional outcomes at the long term	I (32)	3W	NA	NA	NA	NA	NA	NA	95,5 (3,4)
			C (31)	NA							89,32 (5,6)
2	Failla et al. (2016) 5	Preoperative rehabilitation (progressive strengthening and neuromuscular training) had greater functional outcomes and RTS rates 2 years after ACLR	I	NA	88,52	94 (10)	89 (12)	98 (5)	85,6 (18)	76 (20)	NA
			C	NA	73,32	78 (33)	72 (32)	82 (34)	70,6 (33)	64 (32)	
3	Grindem et al. (2015) 9	Progressive pre operative rehab showed superior patient reported outcomes at 2 years postoperatively	I	6M	88,88	93,5 (10,3)	89,2 (11,9)	98 (5,6)	85,1 (16,2)	78,6 (20,4)	NA
			C	NA	78,24	86 (15,1)	77,4 (18)	92,5 (12,8)	67,6 (25,9)	67,7 (22,7)	
4	Reddy et al. (2020) 11	Pre op rehab improve early functional outcome at 3 weeks and 6 weeks	I (20)	3W	NA	NA	NA	NA	NA	NA	93,86
			C (21)	NA							92,43
5	Aiyanna, et al. (2022) 12	Pre op rehab enhance post op outcome as measured up to 1 year.	I (15)	3M	91,87	NA	NA	NA	NA	NA	NA
			C (15)	NA	78,67						

**Abbreviations: C** , Control; **I** , Intervention; **M** , Months; **NA** , Not Available; **W** , Weeks.

From the data gathered from included studies, there are two main outcomes representing patients' subjective functional outcomes observed, the first one is KOOS score and the second one is Lysholm score. The timing of these two are also different where Lysholm score analyze short term functional outcome on three months post operative period, while KOOS score analyze long term outcome on two years post operative period. KOOS score subscales were separated to highlight which subgroup affected by pre operative rehabilitation the most.

KOOS are divided into five subscales, which in total encompases 42 items that represents patients opinion and associated problems. These five subscales are pain, other symptoms, activity of daily living, sports and recreations, and knee related quality of life.

**Fig. 2:**
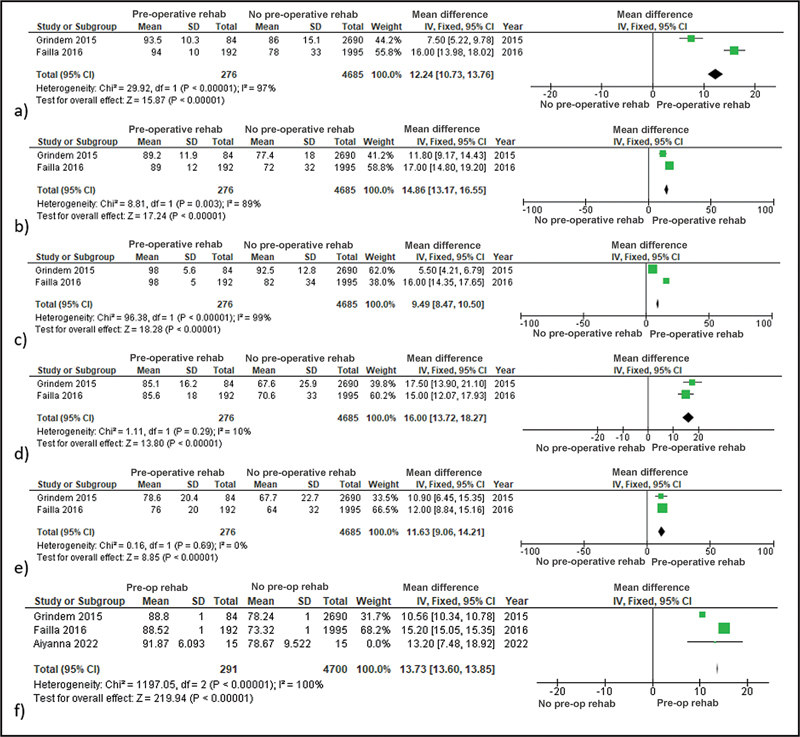
a) Forest Plot of Pain Subscale of the KOOS Score; b) Forest Plot of Symptoms Subscale of the KOOS Score; c) Forest Plot of ADL Subscale of the KOOS Score; d) Forest Plot of Sports and Recreation Subscale of the KOOS Score; e) Forest Plot of QoL Subscale of the KOOS Score; f) Forest Plot of the Total Mean of the KOOS Score.

This meta-analysis analyze 2 years post-operative KOOS score, which divided into 5 subscales and 1 mean of the KOOS score.Pain subscale explains knee and pain related to their knees. In the pain subscale, pre-operative rehabilitation significantly affect the patients' outcome (P < 0.00001, I2: 97%, MD: 12.24) ( Fig. 2a ). The symptoms subscale explains other symptoms of patient's knee that other subscale failed to mention. It is shown that pre operative rehabilitation significantly affect the patients' outcome (p < 0.00001, I2: 89%, MD: 14.86) ( Fig. 2b ). The ADL subscale explains ADL of the patients, especially activities that involve their knee. It is shown that pre operative rehabilitation significantly affect the patients' outcome (p < 0.00001, I2: 99%, MD: 9.49) ( Fig. 2c ). This subscale explains the sports and recreational function of patients' knee. It is concluded that pre operative rehabilitation significantly affect the patients' outcome (p < 0.00001, I2: 10%, MD: 16) ( Fig. 2d ). This QoL measures the quality of living of patients involving their knee function. It is concluded that pre operative rehabilitation significantly affect the patients' outcome (p < 0.00001, I2: 0%, MD: 11.63) ( Fig. 2e ). The total or mean KOOS score represents whole knee function of the patient. It is inferred that pre operative rehabilitation significantly affect the patients' outcome (p < 0.00001, I2: 100%, MD: 13.73) ( Fig. 2f ).

**Fig. 3:**
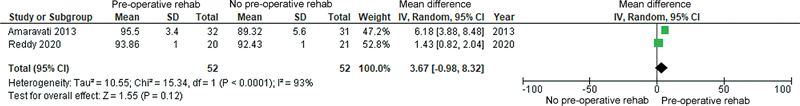
Forest Plot for 3 month post-operative Lysholm Score.

The forest plot below analyses short term outcome of 3 months post ACL reconstruction ( Fig. 3 ). The parameter used to analyze patients' knee function is the Lysholm score. It is shown that pre operative rehabilitation does aid post operative knee function in the short term period, however it doesn't show statistical signicance as good as the long term assessment (p = 0.12, I2: 93%, MD: 3.67).

## Discussion

It has been inferred from multiple studies that pre-operative exercise aids in the outcome of ACL reconstructions. Pre-operative rehabilitation and exercise prepare the patient for surgery physically and mentally. 13 Pre-operative rehabilitation is believed to be able to enhance recovery of the patients due to the preservation of muscle strength and neuromuscular habitation through exercise and other exercises. 5 14 The three muscle group needs to be concentrated regarding the post-operative outcome of ACL surgeries are quadriceps, hamstrings, and gluteal. 14 In a more microscopic and biomolecular level of muscle properties in post ACL reconstruction patients, Shaarani et al. 6 found that muscle cross sectional area are bigger in pre-operative rehabilitation group. Genes contributing to muscle atrophy, MuRF-1, decreased significantly after rehabilitation. 6 Our research conclude that pre-operative rehabilitation improve patients's post-operative outcome through KOOS score.

Research by Kim et al. 15 also conclude that pre-operative rehabilitation and exercise does strengthen lower extremity muscles on their post-operative state. Logerstedt et al. 16 found that quadriceps strength correlates with IKDC 2000 scores in 6 months post ACL reconstruction, hence concluding pre-operative quadriceps strengthening has positive effect on post-operative functional outcome. Regarding muscular activity imbalance of lower extremity function, Ficek et al. 14 shows that pre-operative rehabilitation combined with post-operative rehabilitation significantly reduced imbalance of activity especially in hamstring and gluteal muscle group.

There's only one systematic review which suggests pre-operative rehabilitation contributes to only small portion to improve patients' short-term outcome in ACL reconstruction. 13 Pre-operative rehabilitation aid patients in long term outcome of ACL reconstruction much more compared to short term outcome. As seen in our meta-analysis, where significance are observed on two-years post-operative period, while insignificant outcome improvement are seen on three-months post-operative period.

Multiple protocols regarding pre-operative rehabilitation does concentrate on muscular strength of the patients. Grindem et al. 9 suggests that the goal of pre-operative rehabilitation is to gain 90% of quadriceps and hamstrings strength, as well as hopping performance. This goal is achieved by multiple rehabilitation method such as heavy resistance strength training, plyometrics and neuromuscular exercises, while initiated as soon as joint effusion and ROM deficits were resolved.

Post-operative rehabilitation regimen has been widely used with varieties of methods and goals. Grindem et al. 9 divide post-operative rehabilitation in three phases, first phase (0-2 months) aims to eliminate effusion, restore ROM, and prevent muscular atrophy. This is achieved by applying daily quadriceps contractions, ROM exercises, and cycling as tolerated by the patients. The second phase (2-6 months) aims to regain full control of weight beaing during knee extension, at least 80% of muscle strength, and the ability to hop. The last phase aims to regain at least 90% muscle strength and hopping ability, while transitioning to sports. Regarding the duration of physical therapy, all included studies mention variable duration of rehabilitaion while Failla et al. 5 didn't mention the duration ( Table 4 ). Further research may be needed to determine the optimal rehabilitation duration for ACL injured patient before having their reconstruction.

## Conclusion

Multiple researches have advocated in applying pre-operative rehabilitation in ACL injured patients who will be treated by ACL reconstruction. In multiple parameters such as muscle strength, surface area, atrophic gene expression, and functional scores (IKDC, Lysholm, and KOOS score), the outcome of patients undergoing pre-operative rehabilitation is much more favorable significantly, compared to patients who did not attend a pre-operative rehabilitation program. In our study, the authors concluded that short-term outcome may not be that much significant, however in all KOOS sub group, pre-operative rehabilitation significantly improve the functional outcome of ACL reconstruction procedure.

Through this research, the authors recommend adding a recommendation of pre-operative rehabilitation in ACL injury management protocol to improve patients' outcomes. Further research may be needed to decide a standardised protocol of rehabilitation that may improve patients' undergoing ACL reconstruction outcome.
