# Supplementary material for: Functional Outcome of ACL Reconstruction Following Pre-reconstruction Rehabilitation vs. None Rehabilitation: A Systematic Review and Meta-analysis
Source: Rev Bras Ortop (Sao Paulo). 2024 Apr 10;59(2):e172–9. doi: 10.1055/s-0044-1779327 (PMC11007596; doi:10.1055/s-0044-1779327)
Supplement: Related texts

# Research article: Resultado funcional da reconstrução do LCA após habilitação pré-reconstrução vs. nenhuma pré-habilitação: Revisão sistemática e metanálise

**Keywords:** lesões do ligamento cruzado anterior, reabilitação pré-operatória, reconstrução do ligamento cruzado anterior

## Abstract

**Objetivo** O objetivo deste estudo é analisar as necessidades de pré-habilitação cirúrgica em pacientes submetidos à reconstrução do LCA.

**Métodos** Os relatórios das bases de dados foram pesquisados entre 2018 e 2023, utilizando PubMed, base de dados da biblioteca Cochrane, Medline e outros artigos publicados. Uma análise estatística foi feita utilizando-se o Review Manager.

**Resultados** O grupo de pré-habilitação cirúrgica apresenta pontuação KOOS pós-operatória de 2 anos significativamente maior em todos os subescores e na média total da pontuação, dor (p < 0,0001), sintomas (p < 0,0001), AVD (p < 0,0001). 0,0001), esportes e recreação (p < 0,0001), QV (p < 0,0001) e média total do escore KOOS (p < 0,0001). Em contrapartida, o grupo de habilitação pré-operatória apresenta pontuação insignificantemente maior no escore de Lysholm pós-operatório de 3 meses (p = 0,12).

**Conclusão** Esta metanálise conclui que a pré-habilitação cirúrgica pode proporcionar melhores resultados pós-operatórios a longo prazo, no entanto, pode não proporcionar um resultado significativo a curto prazo. Recomenda-se adicionar a pré-habilitação cirúrgica como uma diretriz para o manejo da lesão do LCA para melhorar o resultado a longo prazo dos pacientes com lesão do LCA submetidos ao procedimento de reconstrução do LCA.

## Introdução

A reconstrução do LCA continua sendo a base do tratamento para rupturas do LCA em todo o mundo. A taxa de reconstrução mantém uma tendência consistente com o número crescente de rupturas do LCA, 1 2 podendo chegar a 68,6 casos por 100.000 pessoas anualmente. 3

Ultimamente, uma população mais ativa e mais jovem tem sido afetada, gerando uma necessidade de recuperação mais rápida e recuperação de funções ótimas do joelho. 4 Profissionais visam obter as melhores estratégias para o manejo de casos de ruptura do LCA. 5

Embora possa parecer insignificante, um programa de pré-habilitação serve como um excelente plano de tratamento complementar, melhorando o resultado da reconstrução do LCA. 6 Eitzen et al. 7 mencionaram que a força muscular do quadríceps pré-operatória produz resultados funcionais melhores no joelho. Em contraste com o grupo sem pré-habilitação cirúrgica, que possui resultados funcionais menos satisfatórios. 8 Este estudo tem como objetivo responder à pergunta "a pré-habilitação é necessária para melhorar o resultado da reconstrução do LCA?" comparando a pré-habilitação pré-reconstrução com a não pré-habilitação em relação ao resultado funcional da reconstrução do LCA através de uma publicação recente.

## Métodos

Esta revisão sistemática e metanálise foram realizadas seguindo as diretrizes PRISMA, lista de verificação QUOROM e diagrama de fluxo para metanálise para ensaios clínicos randomizados (ECR).

## Estratégia de pesquisa e critérios de seleção

Realizamos uma pesquisa sistemática na literatura em inglês no PubMed/MEDLINE, ZETOC, EMBASE, AMED, CINAHL, Cochrane Central Register of Controlled Trials (CENTRAL) e ClinicalTrials.gov, publicada de janeiro de 2018 a janeiro de 2023. Os termos de pesquisa incluem, mas não estão limitados a “pre-reconstruction rehabilitation”, “pre-operative rehabilitation”, “pre-operative exercise”, “pre-rehabilitation”, “none rehabilitation”, “anterior cruciate ligament”, “ACL rupture”, “ACL tear”, “treatment”, “quality of life”, “functional score”, “KOOS”.

Todos os tipos de ensaios clínicos randomizados, coortes e séries de casos publicados como artigo completo foram incluídos neste estudo. As peças foram selecionadas com base nos critérios de inclusão e exclusão declarados de acordo com o método PICO (Population, Intervention, Comparison, Outcome), conforme descrito na Tabela 1 .

**Tabela 1:** Tabela PICO para Critérios de Inclusão e Exclusão

	Critérios de inclusão	Critérios de exclusão
**População**	• De 18 a 40 anos • Planejado para cirurgia reconstrutiva • Participar de reabilitação pré-operatória	• Idade < 18 ou >40 • Ruptura aguda do LCA • Lesão complexa
**Intervenção**	Programa de fisioterapia pré-reconstrução.	• Os pacientes são submetidos a tratamento conservador
**Procedimento Padrão**	Somente reabilitação pós-operatória Nenhum programa de reabilitação	
**Outcome**	Pontuação KOOS Pontuação de Lysholm	• Medidas de resultados não relatadas na conclusão. • As medidas de resultados apenas reportam a diferença. • Medidas de resultados recolhidas em diferentes. • As medidas de resultados não são comparáveis entre si
**Desenho de pesquisa**	Ensaio randomizado controlado, coorte, série de casos	Relato de Caso, Revisão Sistemática, Metanálise

## Extração de dados

Os dados foram extraídos por meio de um formulário padronizado de coleta de dados por uma equipe de pesquisa, com cada artigo escolhido selecionado de forma independente por dois revisores. As divergências entre os revisores sobre a inclusão ou exclusão de um estudo foram resolvidas por consenso e consulta a um terceiro revisor, quando necessário. As variáveis coletadas incluem a pontuação KOOS e a pontuação Lysholm.

## Análise de dados

Os dados extraídos foram avaliados quanto à heterogeneidade clínica. Devido às diferenças nas intervenções de exercício investigadas, nas populações estudadas e nas medidas de resultados, concluiu-se que os estudos incluídos não eram homogêneos. Portanto, uma metanálise não pôde ser realizada. A análise dos dados e o gráfico florestal foram gerados utilizando o Review Manager (RevMan), da Cochrane Collaboration, 2020.

## Avaliação de Qualidade

Os estudos incluídos foram avaliados em termos de qualidade por dois revisores independentes com base nos 13 itens da Diretriz de Método Atualizado para Revisões Sistemáticas de 2015 no Cochrane Back and Neck Group.

## Resultados

O processo de seleção dos estudos é apresentado no fluxograma PRISMA. Foram identificados 206 registros por meio de buscas em bancos de dados, enquanto registros adicionais identificados por outras fontes totalizaram 174. Duplicatas de 63 estudos foram removidas. Os registros foram avaliados pela equipe de pesquisa e 125 registros foram excluídos. Foram avaliados os artigos completos para a elegibilidade de 29 artigos. Artigos completos foram excluídos devido ao desenho do estudo ou ponto de coleta de dados. Portanto, 5 estudos foram incluídos na síntese qualitativa ( Fig. 1 ). Os estudos incluídos são apresentados na Tabela 2 . 5 9 10 11 12 As características populacionais dos estudos incluídos são apresentadas na Tabela 3 5 9 10 11 12 enquanto as características dos resultados dos estudos incluídos são apresentadas na Tabela 4 . 5 9 10 11 12

**Fig. 1:** Triagem de pesquisa guiada pelo fluxograma PRISMA.

**Tabela 2:** Estudos incluídos na metanálise

N°	Autor (ano)	Revista	Desenho de pesquisa	Nível de evidência
**1**	Amaravati et al. (2013) 10	ISAKOS	ECR prospectivo cego simples	2a
2	Failla et al. (2016) 5	American Journal of Sports Medicine	Coorte	3
3	Grindem et al. (2015) 9	British Journal of Sports Medicine	Coorte	2a
4	Reddy et al. (2020) 11	Ambulatory Surgery	estudo prospectivo randomizado	2a
5	Aiyanna, et al. (2022) 12	International Journal of Recent Scientific Research	estudo prospectivo	2a

**Tabela 3:** Características Populacionais dos Estudos

N°	Autor (ano)	Intervenção	Procedimento	Tamanho da amostra (n)	Gênero (masculino/feminino)	Idade (anos)	Acompanhamento (meses)
1	Amaravati et al. (2013) 10	R	SR	I: 32	NA	NA	ROM: 6s, 3m, 6m, 1a
				C: 31			Dor, Grith, Lysholm, atividade de Tegner, LEFS, IKDC, teste de salto
2	Failla et al. (2016) 5	R	SR	I: 192	Masculino 55%	24,3 ± 10	IKDC e KOOS pré e 2 anos pós
				C: 1995			
3	Grindem et al. (2015) 9	R	SR	I: 84	Masculino 54%	24,7 ± 9	KOOS pré-operatório (pós-reabilitação), 2 anos pós-operatório
				C: 2690			
4	Reddy et al. (2020) 11	R	SR	I: 20	M: 38	I 27,48	Grau IKDC, Lysholm, ROM pré, 3s, 6s, 3m, 6m pós-operatório
				C: 21	F: 3	C 28,17	
5	Aiyanna, et al. (2022) 12	R	SR	I: 15	M: 25	0-20: (27%)20-40: (67%)40-60 (6%)	Pontuação IKDC, KOOS, Lysholm 3, 6 e 12 meses
				C: 15	F: 5		

**Abreviações: C** , Controle; **I** , Intervenção; **IKDC** , International Knee Documentation Committee; **KOOS** , Knee Injury and Osteoarthritis Outcome Score; **LEFS** , Lower extremity functional scale; **SR** , Sem reabilitação; **R** , reabilitação; **ROM** , amplitude de movimento.

**Tabela 4:** Características dos resultados dos estudos

N°	Autor (ano)	Conclusão	Grupo (N)	Período de reabilitação	KOOS Média	KOOS Dor	KOOS Sintomas	KOOS AVD	KOOS Esportes	KOOS QV	Pontuação de Lysholm
1	Amaravati et al. (2013) 10	O exercício pré-operatório resultou em melhores resultados funcionais pós-operatórios a longo prazo	I (32)	3S	NA	NA	NA	NA	NA	NA	95,5 (3,4)
			C (31)	NA							89,32 (5,6)
2	Failla et al. (2016) 5	A reabilitação pré-operatória (fortalecimento progressivo e treinamento neuromuscular) teve maiores resultados funcionais e taxas de RTS 2 anos após RLCA	I	NA	88,52	94 (10)	89 (12)	98 (5)	85,6 (18)	76 (20)	NA
			C	NA	73,32	78 (33)	72 (32)	82 (34)	70,6 (33)	64 (32)	
3	Grindem et al. (2015) 9	A reabilitação pré-operatória progressiva mostrou resultados superiores relatados pelos pacientes aos 2 anos de pós-operatório	I	6m	88,88	93,5 (10,3)	89,2 (11,9)	98 (5,6)	85,1 (16,2)	78,6 (20,4)	NA
			C	NA	78,24	86 (15,1)	77,4 (18)	92,5 (12,8)	67,6 (25,9)	67,7 (22,7)	
4	Reddy et al. (2020) 11	A reabilitação pré-operatória melhora o resultado funcional precoce em 3 e 6 semanas	I (20)	3s	NA	NA	NA	NA	NA	NA	93,86
			C (21)	NA							92,43
5	Aiyanna, et al. (2022) 12	A reabilitação pré-operatória melhora o resultado pós-operatório medido em até 1 ano.	I (15)	3m	91,87	NA	NA	NA	NA	NA	NA
			C (15)	NA	78,67						

**Abreviaturas:** C, Controle; I, Intervenção; M, Meses; NA, não disponível; S, semanas.

A partir dos dados coletados nos estudos incluídos, existem dois principais dados que representam os resultados funcionais subjetivos dos pacientes, sendo o primeiro a pontuação KOOS e o segundo a pontuação de Lysholm. O momento de análise destes é também diferente, onde a pontuação de Lysholm avalia o resultado funcional a curto prazo, três meses após a cirurgia, enquanto a pontuação KOOS avalia o resultado a longo prazo, dois anos após a cirurgia. As subescalas da pontuação KOOS foram separadas para destacar qual subgrupo foi mais afetado pela pré-habilitação operatória.

As pontuações do KOOS estão divididas em cinco subescalas, que no total abrangem 42 itens que representam a opinião dos pacientes e os problemas associados. Essas cinco subescalas são dor, outros sintomas, atividades da vida diária, esportes e recreação, e qualidade de vida relacionada ao joelho.

Esta metanálise analisa a pontuação KOOS pós-operatória de 2 anos, que se divide em 5 subescalas e 1 média da pontuação KOOS. A subescala de dor explica o joelho e a dor relacionada aos joelhos. Na subescala dor, a pré-habilitação cirúrgica afeta significativamente o resultado dos pacientes (P < 0,00001, I2: 97%, DM: 12,24) ( Fig. 2a ). A subescala de sintomas explica outros sintomas do joelho do paciente que outras subescalas não mencionaram. É demonstrado que a pré-habilitação cirúrgica afeta significativamente o resultado dos pacientes (p < 0,00001, I2: 89%, DM: 14,86) ( Fig. 2b ). A subescala AVD explica as AVD dos pacientes, principalmente atividades que envolvem o joelho. É demonstrado que a pré-habilitação cirúrgica afeta significativamente o resultado dos pacientes (p < 0,00001, I2: 99%, DM: 9,49) ( Fig. 2c ). Esta subescala explica a função desportiva e recreativa do joelho dos pacientes. Conclui-se que a pré-habilitação cirúrgica afeta significativamente o resultado dos pacientes (p < 0,00001, I2: 10%, DM: 16) ( Fig. 2d ). A QV mede a qualidade de vida dos pacientes envolvendo a função do joelho. Conclui-se que a pré-habilitação cirúrgica afeta significativamente o resultado dos pacientes (p < 0,00001, I2: 0%, DM: 11,63) ( Fig. 2e ). A pontuação KOOS total ou média representa toda a função do joelho do paciente. Infere-se que a pré-habilitação cirúrgica afeta significativamente o resultado dos pacientes (p < 0,00001, I2: 100%, DM: 13,73) ( Fig. 2f ).

**Fig. 2:** a) Gráfico florestal da subescala de dor da pontuação KOOS; b) Gráfico Florestal da subescala de sintomas da pontuação KOOS; c) Gráfico Florestal da Subescala de Atividades da Vida Diária (ADL) da Pontuação KOOS; d) Gráfico Florestal da Subescala de Esportes e Recreação da Pontuação KOOS; e) Gráfico Florestal da Subescala de Qualidade de Vida (QV) da Pontuação KOOS; f) Gráfico Florestal da Média Total da Pontuação KOOS.

O gráfico florestal abaixo analisa o resultado no curto prazo de 3 meses após a reconstrução do LCA ( Fig. 3 ). O parâmetro utilizado para analisar a função do joelho dos pacientes é o escore de Lysholm. É demonstrado que a pré-habilitação cirúrgica ajuda a função pós-operatória do joelho no curto prazo, porém não mostra significância estatística tão boa quanto a avaliação a longo prazo (p = 0,12, I2: 93%, DM: 3,67).

**Fig. 3:** Gráfico Florestal para a Pontuação de Lysholm 3 meses após a operação.

## Discussão

Foi inferido de vários estudos que a fisioterapia pré-cirúrgica auxilia no resultado das reconstruções do LCA. A pré-habilitação cirúrgica e os exercícios preparam o paciente para a cirurgia física e mentalmente. 13 Acredita-se que a pré-habilitação cirúrgica seja capaz de melhorar a recuperação dos pacientes devido à preservação da força muscular e adaptação neuromuscular por meio de exercícios e outras atividades. 5 14 Os três grupos musculares que precisam ser focados em relação ao resultado pós-operatório das cirurgias do LCA são quadríceps, isquiotibiais e glúteos. 14 Em um nível mais microscópico e biomolecular das propriedades musculares em pacientes pós-reconstrução do LCA, Shaarani et al. 6 descobriram que a área de secção transversa muscular é maior no grupo de pré-habilitação operatória. Os genes que contribuem para a atrofia muscular, MuRF-1, diminuíram significativamente após a fisioterapia. 6 Nossa pesquisa conclui que a pré-habilitação cirúrgica melhora o resultado pós-operatório dos pacientes por meio do escore KOOS.

O estudo de Kim et al. 15 também conclui que a reabilitação e os exercícios pré-operatórios fortalecem os músculos das extremidades inferiores no estado pós-operatório. Logerstedt et al. 16 descobriram que a força do quadríceps se correlaciona com os escores do IKDC 2000 em 6 meses após a reconstrução do LCA, concluindo assim que o fortalecimento pré-operatório do quadríceps tem efeito positivo no resultado funcional pós-operatório. Em relação ao desequilíbrio da atividade muscular da função dos membros inferiores, Ficek et al. 14 mostram que a pré-habilitação cirúrgica combinada com a reabilitação pós-operatória reduziu significativamente o desequilíbrio da atividade, especialmente nos grupos musculares isquiotibiais e glúteos.

Há apenas uma revisão sistemática que sugere que a pré-habilitação cirúrgica contribui apenas em pequena parte para melhorar o resultado a curto prazo dos pacientes na reconstrução do LCA. 13 A pré-habilitação cirúrgica ajuda os pacientes muito mais no resultado de longo prazo da reconstrução do LCA do que no curto prazo. Como visto em nossa metanálise, onde são observadas significâncias no período pós-operatório de dois anos, enquanto melhorias insignificantes nos resultados são observadas no período pós-operatório de três meses.

Vários protocolos de reabilitação pré-operatória concentram-se na força muscular dos pacientes. Grindem et al. 9 sugere que o objetivo da pré-reabilitação operatória é ganhar 90% da força do quadríceps e isquiotibiais, bem como desempenho de salto. Este objetivo é alcançado por múltiplos métodos de fisioterapia, como treinamento de força de resistência pesada, pliometria e exercícios neuromusculares, embora iniciados assim que o derrame articular e os déficits de ADM forem resolvidos.

O regime de reabilitação pós-operatória tem sido amplamente utilizado com diversos métodos e objetivos. Grindem et al. 9 divide a reabilitação pós-operatória em três fases, a primeira fase (0-2 meses) visa eliminar a efusão, restaurar a ADM e prevenir a atrofia muscular. Isto é conseguido aplicando contrações diárias do quadríceps, exercícios de ROM e ciclismo conforme tolerado pelos pacientes. A segunda fase (2-6 meses) visa recuperar o controle total do peso durante a extensão do joelho, pelo menos 80% da força muscular e a capacidade de pular. A última fase visa recuperar pelo menos 90% da força muscular e da capacidade de salto, durante a transição para o esporte. Em relação ao tempo de fisioterapia, todos os estudos incluídos mencionam duração variável da reabilitação, enquanto Failla et al. 5 não mencionaram a duração ( Tabela 4 ). Mais pesquisas podem ser necessárias para determinar a duração ideal da reabilitação para pacientes com lesão do LCA antes de serem reconstruídos.

## Conclusão

Diversas pesquisas têm defendido a aplicação da pré-reabilitação operatória em pacientes com lesão do LCA que serão submetidos a reconstrução do LCA. Em diversos parâmetros, como força muscular, área superficial, expressão gênica atrófica e pontuações funcionais (IKDC, Lysholm e pontuação KOOS), os resultados de pacientes submetidos à pré-reabilitação operatória são significativamente mais favoráveis em comparação com pacientes que não participaram de um programa de reabilitação pré-operatória. Em nosso estudo, os autores concluíram que o resultado a curto prazo pode não ser tão significativo, porém em todos os subgrupos do KOOS, a pré-reabilitação operatória melhora significativamente o resultado funcional do procedimento de reconstrução do LCA.

Com base nesta pesquisa, os autores recomendam a inclusão da recomendação de reabilitação pré-operatória no protocolo de manejo de lesões do LCA para melhorar os resultados dos pacientes. Pesquisas adicionais podem ser necessárias para definir um protocolo padronizado de reabilitação que possa aprimorar os resultados dos pacientes submetidos à reconstrução do LCA.
